# Standardising practices improves ambulatory diabetic foot management and reduces amputations: the Queensland Diabetic Foot Innovation Project, 2006 – 2009

**DOI:** 10.1186/1757-1146-4-S1-O25

**Published:** 2011-05-20

**Authors:** Peter A Lazzarini, Sharon R O’Rourke, Anthony Russell, Patrick H Derhy, Maarten C Kamp

**Affiliations:** 1 Allied Health Research Collaborative, Metro North Health Service District, Queensland Health, Brisbane, Australia; 2 Department of Podiatry, Metro North Health Service District, Queensland Health, Brisbane, Australia; 3 School of Public Health, Queensland University of Technology, Brisbane, Australia; 4 Cairns Diabetes Centre, Queensland Health, Cairns, Australia; 5 Department of Diabetes & Endocrinology, Princess Alexandra Hospital, Brisbane, Australia; 6 Diamantina Institute, The University of Queensland, Brisbane, Australia; 7 Centre for Healthcare Improvement, Queensland Health, Brisbane, Australia; 8 School of Medicine, The University of Queensland, Brisbane, Australia; 9 Department of Endocrinology, Metro North Health Service District, Queensland Health, Brisbane, Australia

## Background

Diabetic foot complications are recognised as the most common reason for diabetic related hospitalisation and lower extremity amputations. Multi-faceted strategies to reduce diabetic foot hospitalisation and amputation rates have been successful. However, most diabetic foot ulcers are managed in ambulatory settings where data availability is poor and studies limited. The project aimed to develop and evaluate strategies to improve the management of diabetic foot complications in three diverse ambulatory settings and measure the subsequent impact on hospitalisation and amputation.

## Methods

Multifaceted strategies were implemented in 2008, including: multi-disciplinary teams, clinical pathways and training, clinical indicators, telehealth support and surveys. A retrospective audit of consecutive patient records from July 2006 – June 2007 determined baseline clinical indicators (n = 101). A clinical pathway teleform was implemented as a clinical record and clinical indicator analyser in all sites in 2008 (n = 327) and followed up in 2009 (n = 406).

## Results

Prior to the intervention, clinical pathways were not used and multi-disciplinary teams were limited. There was an absolute improvement in treating according to risk of 15% in 2009 and surveillance of the high risk population of 34% and 19% in 2008 and 2009 respectively (p < 0.001). Improvements of 13 – 66% (p < 0.001) were recorded in 2008 for individual clinical activities to a performance > 92% in perfusion, ulcer depth, infection assessment and management, offloading and education. Hospitalisation impacts recorded reductions of up to 64% in amputation rates / 100,000 population (p < 0.001) and 24% average length of stay (p < 0.001)

## Conclusion

These findings support the use of multi-faceted strategies in diverse ambulatory services to standardise practice, improve diabetic foot complications management and positively impact on hospitalisation outcomes. As of October 2010, these strategies had been rolled out to over 25 ambulatory sites, representing 66% of Queensland Health districts, managing 1,820 patients and 13,380 occasions of service, including 543 healed ulcer patients. It is expected that this number will rise dramatically as an incentive payment for the use of the teleform is expanded.

